# Antibacterial, Antioxidant Potency, and Chemical Composition of Essential Oils from Dried Powdered Leaves and Flowers of *Hypericum revolutum* subsp. *keniense* (Schweinf.)

**DOI:** 10.1155/2023/4125885

**Published:** 2023-01-03

**Authors:** Geoffrey Ogeto Sengera, Evans Okemwa Kenanda, Jared Misonge Onyancha

**Affiliations:** ^1^Department of Pharmacy, Kisii University, P. O. Box 408-40200, Kisii, Kenya; ^2^Department of Research and Extension, Kisii University, P. O. Box 408-40200, Kisii, Kenya; ^3^Department of Pharmacognosy, Mount Kenya University, P.O. BOX 342-0100, Thika, Kenya

## Abstract

*Hypericum revolutum* subsp. *keniense* is a plant mainly used to treat diarrhoea, rheumatism, nervous disorders, and wounds in African traditional medicine. The objective of the current work was to establish antibacterial, antioxidant potency, and chemical composition of essential oil from the leaves and flowers of *Hypericum revolutum* subsp. *keniense*. The oils were isolated by steam distillation. Antibacterial activity against methicillin-resistant *Staphylococcus aureus*, *Staphylococcus aureus* (ATCC 12393)*, Escherichia coli* (ATCC 25922), *Acinetobacte*r *baumannii* (ATTC 19606), *Salmonella enteritidis* (NCTC12023), *Salmonella typhimurium* (ATCC 14028), *Pseudomonas aeruginosa* (ATCC 15442), and *Haemophilus influenzae* (ATCC 49766) was carried out by agar disk diffusion and microtiter broth dilution methods. Antioxidant activities of the essential oils were examined by different methods, DPPH, FRAP, and H_2_O_2_ assays. Chemical characterization was performed using gas chromatography interfaced with mass spectrometry, Fourier-transform infra-red (FTIR) spectroscopy, and quantification of phenolics and flavonoids by Folin–Ciocalteu reagent and aluminium nitrate, respectively. The oils showed potential antibacterial activity with mean zone of inhibition ranging from 20.67 ± 0.33 to 32.00 ± 1.00 mm at 100% oil concentration against the tested bacteria. Furthermore, the minimum inhibitory concentrations (MICs) in all the tested microorganisms were in the range from 250 to 15.6250 *μ*g/ml. The essential oils derived from the leaves revealed varied antioxidant activity levels with the different methods of assay. The IC_50_ of values obtained from the three methods, DPPH, FRAP, and H_2_O_2_ were ˃1000 *μ*g/ml, 0.31 *μ*g/ml, and 12.33 *μ*g/ml, respectively. Caryophyllene (22.1%) and 2, 3, 4-trimethylhexane were the major chemical components of the essential oils derived from the leaves and flowers, respectively. FTIR spectroscopy of the essential oils from the leaves and flowers showed similarity at peaks for hydroxyl, unsaturated olefinic, and amine functional groups. Further findings indicated that the total phenolic and flavonoid contents essential oils derived from leaves were 130.4 6 ± 10.5 mg GAE/g dry weight and 0.911 ± 0.04 mg CE/g dry weight, respectively. It was therefore concluded that essential oils from the leaves and flowers of *H. revolutum* subsp. *keniense* have compounds that have antibacterial and antioxidant potency.

## 1. Introduction

Essential oils are mainly used as antimicrobial agents [1]. Also, they are used as insecticides, insect repellents, antimalaria, antidepressant, anticancer, antimutagenic, anti-inflammatory, analgesic, antipyretics, antioxidant, anticonvulsants, conjunctivitis, tuberculosis, stress, bronchitis, anxiety, neurological disorders, ulcers, liver conditions, and Parkinson's disease [2–4]. The diverse biological activities of essential oils are attributed to the complex nature of chemical composition [2]. The main components of essential oils are monoterpenes and sesquiterpenes, and they exert antibacterial effects by structural and functional damages of the bacterial cell wall [5]. The known antimicrobial activities of essential oils are based on the ability to destroy the cell wall and cell membranes, and they act by causing destabilization of bacterial membranes, damaging the membrane proteins, depleting proton motive force, and releasing cell contents [6]. Essential oils are accumulated mainly by plants as secondary metabolites though a few of them are found in animals and microorganisms [7]. Essential oils are relatively distributed in the plant kingdom in about 60 plant families that comprise close to 2000 species. The major plant families that yield commercially significant essential oils are found mostly in dicotyledonous plants [8]. However, some monocotyledonous and gymnospermatophyta type of plants also produce the essential oils found in the markets [8, 9]. Some essential oils have also been reported in pteridophytes, bryophytes, algae, and fungi too [7].

Plant species of the genus *Hypericum* have a wide scope of secondary metabolites, which are responsible for their many biological activities [10]. Scores of research studies have demonstrated that essential oils have chemical compounds with varied biological and pharmacological properties. In the *Hypericum* genus, *Hypericum perforatum* is the most popular and best studied of all the *Hypericum* species across the world and has been found largely useful in mild to moderate depression, neuroprotection, antineuralgic, and antiviral properties [11]. Also, biological activities such as antibacterial, antioxidant, analgesic, anti-inflammatory, hypotensive, and spasmolytic properties have been reported for *Hypericum perforatum* [12]. The other *Hypericum* species have demonstrated antioxidant, antimicrobial, anti-inflammatory, cytotoxic, antimalaria, analgesic, wound, and burn healing properties [12, 13], and out of the various biological activity studies of the essential oils from *Hypericum* species, antimicrobial activities are the most reported [13, 14].

The species *Hypericum revolutum* subsp. *keniense* is used in traditional medicine to treat skin disorders, nervous disorders, gastrointestinal disorders, and rheumatism [15, 16]. Limited works on biological, pharmacological, and phytochemical studies of *Hypericum revolutum* subsp. *keniense* have been reported for the organic and aqueous extracts, that is, antimicrobial, brine shrimp lethality, and oral acute toxicity and preliminary phytochemical studies that revealed the presence of saponins, cardiac glycosides, flavonoids, tannins, coumarins, carotenoids, and volatile oils [17] and isolation of betulinic acid and coumarins [17, 18]. To the best of our knowledge, there are no reports on the biological or pharmacological activities of the essential oils from *Hypericum revolutum* subsp. *keniense*. The objective of the current study was to establish the antibacterial and antioxidant potency and characterize the composition of the essential oil components from this plant. The findings from this study imperatively provide scientific data to validate the ethnomedicinal claims of using the plant extracts for the management of gastrointestinal, skin, and musculoskeletal disorders.

## 2. Materials and Methods

### 2.1. Collection of Plant Materials

The fresh leaves and flowers of *Hypericum revolutum* subsp. *keniense* were collected from Kieni village, Gakoe sublocation in Kiambu County. The plant was authenticated by a taxonomist at the East Africa Herbarium of the National Museums of Kenya. A voucher specimen number GS001-2020 was prepared and deposited at the Department of Botany in the East Africa Herbarium. The collected leaves and flowers were air dried at room temperature and pressure for two weeks. The dried plant materials were ground into course powder using a blender.

### 2.2. Extraction of Essential Oil from Leaves and Flowers of *H. revolutum* subsp. *keniense*

The leaves and flower powders of *H. revolutum* subsp. *keniense* (500 g) were extracted using Clevenger-like apparatus for 2 hours according to the British pharmacopoeia steam distillation method [19]. The essential oils that were extracted were collected and dried over anhydrous sodium sulphate; thereafter, the oils were weighed and stored in airtight sealed vials at 4°C until antibacterial and GC-MS analysis of the essential oils was conducted.

### 2.3. Microorganisms Used for Antibacterial Assay

The microorganisms that were used in this study were American type culture collections (ATCCs) and clinical (Laboratory collections). The ATCC microorganisms *Staphylococcus aureus* (ATCC 12393)*, Escherichia coli* (ATCC 25922), *Acinetobacte*r *baumannii* (ATTC 19606), *Salmonella enteritidis* (NCTC12023), *Salmonella typhimurium* (ATCC 14028), and *Pseudomonas aeruginosa* (ATCC 15442) were obtained from Kenya Medical Research Institute (KEMRI) laboratory. In addition, *Haemophilus influenzae* (ATCC 49766) and *Pseudomonas aeruginosa* (ATCC 15442) and a clinical strain of methicillin-resistant *Staphylococcus aureus* were sourced from National Public Health Laboratories Services at Kenyatta Hospital. All bacterial strains were handled and transported aseptically. The bacteria were grown and maintained in small vials containing nutrient broth at 4°C using a Samsung refrigerator (Samsung electronics, Seoul, South Korea) until the time of assay.

### 2.4. Preparation of Culture Media, Plates, Inoculum Bacterial Stains

#### 2.4.1. Mueller Hinton Media and Plates

The Mueller Hinton Agar (MHA) of lot number 0000378040 and Mueller Hinton broth (MHB) of lot number 0000349752 manufactured by HiMedia Laboratories (India) were prepared according to the manufacturer's instruction [20, 21]. About 20 ml of the partially cooled sterile media (45°C) was poured onto the sterile disposable petri dishes to make Mueller Hinton plates with uniform media depth of 3 mm. The Mueller Hinton agar plates had been marks of six equal segments to ensure that a disk was not placed closer than 24 mm (center to center) on the MH agar plate.

#### 2.4.2. Bacteria Inoculum

Each of the eight bacteria used in this study were subcultured overnight at 37°C in Mueller-Hinton agar plates. The direct colony suspension method was then used to prepare bacteria inoculum by transferring five morphologically similar colonies from fresh Mueller Hinton agar plates using a heat sterilized wire loop into about 5 mL of sterile normal saline in a capped test tube and was vortexed. The suspension containing bacterial inoculum was adjusted using sterile normal saline to obtain a turbidity equivalent to that of a 0.5 McFarland standard BaSO_4_ which was prepared spectrophotometrically and therefore an approximate 1.5 × 10^8^ colony forming units per milliliter (CFU/mL) were prepared.

#### 2.4.3. Preparation of Resazurin (Alamar Blue)

Resazurin was obtained from Monks Medicare Africa Ltd in a tablet form. Resazurin solution was prepared according to the manufacturer's specification. A tablet was dissolved in 50 mL of sterilized distilled water and vortex; thereafter, a ratio of 1 : 10 final volume was used for the assay.

### 2.5. Antibacterial Assay

#### 2.5.1. Agar Disk Diffusion Assay

Agar disk diffusion assay was carried out according to the 12^th^ edition of Clinical and Laboratory Standards Institute (CLSI) protocol in 2015 [22] with minor modifications as described in 2016 by Hudzicki [23]. Each bacteria inoculum of approximate 1.5 × 10^8^ CFU/mL was diluted in a ratio of 1 : 100 using sterile normal saline to obtain a colony suspension of 1 × 10^6^ CFU/mL in an inoculum tube. Then, a sterile swab was dipped into an inoculum tube and the swab was rotated against the side of the tube (above the fluid level) using firm pressure to remove excess fluid and swap dripping wet. Thereafter, the dried surface of a MH agar plate was inoculated by streaking the swab three times over the entire agar surface. The plate was rotated approximately 60 degrees each time to ensure an even distribution of the inoculum. Sterile disks (6 mm in diameter) were impregnated with 10 *μ*l of (100%, 50%, 25%, and 12.5% v/v) of the *H. revolutum* subsp. *keniense* leaf and flower essential oils. The impregnated disks were placed on the surface of the agar, using sterilized forceps at a distance of more than 24 mm apart. Experiments for each sample were conducted in triplicates and 10 *μ*l of ciprofloxacin (0.5 mg/ml) was used as positive controls while 10 *μ*l of dimethylsulfoxide (0.1%) was used as a negative control. The inoculated plates containing the essential oil impregnated disks were incubated for 24 hours at 37°C and respective zones of inhibition were measured in mm using the digital zone of inhibition measuring caliper.

#### 2.5.2. Broth Microdilution Assay

Minimum inhibitory concentration (MIC) of *H. revolutum* subsp. *keniense* essential oils was evaluated by the broth microdilution assay according to the procedures described in 2017 by Ohikhena et al. [24] and modified in 2020 by Mogana et al. [25]. First, Mueller Hinton broth (100 *μ*l) was put into the wells from the second to the eleventh column from row B to G. Second, hundred microliters of *H. revolutum* subsp. *keniense* essential oils extracts at concentration 500 *μ*g/mL and the standard drug (ciprofloxacin at concentration 0.8 *μ*g/mL) were added into row B of column 2–9 in quadriplicate using multichannel pipette.

Hundred microliters were removed from the starting concentrations (columns 2–9 in row B) and transferred to the next row and properly mixed with the 100 *μ*l broth that was already in the wells. Two-fold serial dilution from row C to G was performed by transferring 100 *μ*l from row C to row D systematically, followed by mixing gently using microchannel pipette. The procedure was repeated up for row (E), (F), and (G). The last 100 *μ*l that was removed from row G was discarded. The 10^th^ and 11^th^ columns were treated as a negative control which served as growth control (drug-free) columns. Hundred µl of the diluent (1% DMSO in MH broth) were added into 100 *μ*l in row B of column of the 10^th^ and 11^th^ columns. Two-fold serial dilution was performed from row B to row G of the 10^th^ and 11^th^ columns. Finally, the resultant volume in all the test wells with the extracts, the standard drugs, and the negative controls was 100 *μ*l. A volume of 5 *μ*l of the 1 × 10^6^ CFU/ml bacteria inoculum was transferred into all wells except for the wells in the row G which served as a blank control (microorganism and drug-free).

The microtiter plates were incubated at 37°C for 18 hours after which, 60 *μ*l of Alamar blue resazurin was added to all the wells and then incubated for 20 minutes after which the colour change from blue/purple to pink was observed to indicate minimum inhibitory concentration. The viable cells produce oxidoreductases which convert blue resazurin to resorufin (pink) therefore indicating microbial growth and used to determine minimum inhibitory concentration [25, 26].

#### 2.5.3. Determination of Minimum Bactericidal Concentration

Minimum bactericidal concentrations (MBCs) were determined according to the procedure described by Man et al. [27] with slight modifications. Three microliters of Mueller Hinton broth were taken from the last three wells of each row that indicated no bacterial growth and was subcultured in the petri dish plates containing 20 ml fresh Mueller Hinton agar (MHA) and were incubated for 24 h at 37°C. The lowest concentration without visible microbial biomass growth under optical microscope was defined as MBC, indicating the death of 99.5% of the original inoculum.

#### 2.5.4. Disposal of Microbiological Waste

The used petri dishes, 96-micro-titre well plated, and bacteria inoculum were autoclaved before disposing them into the trash.

### 2.6. Investigation of the Antioxidant Activity

#### 2.6.1. DPPH (2, 2-diphenyl-2-picrylhydrazyl) Assay

The antioxidant activity of *Hypericum revolutum* subsp. *keniense* leaf essential oil extract was evaluated using 2, 2-diphenyl-2-picrylhydrazyl (DPPH) free radical scavenging assay method described by Moriasi et al. [28] with slight modifications. Test concentrations of *Hypericum revolutum* subsp. *keniense* leaf essential oil extract and standard (L-ascorbic acid) were prepared at concentrations of 1000 *μ*g/ml, 100 *μ*g/ml, 10 *μ*g/ml, 1 *μ*g/ml, 0.1 *μ*g/ml, and 0.01 *μ*g/ml in methanol. DPPH (0.1 mM) was prepared in methanol and 2.4 ml were added to 1.6 ml of the different concentrations of the extract and the standard L-ascorbic acid. The control for the current study consisted of a mixture of 2.4 ml of (0.1 mM) 2, 2-diphenyl-2-picrylhydrazyl (DDPH) solution in methanol and 1.6 ml of methanol. The test samples and the standard were prepared in triplicate test tubes which were then kept in the dark at room temperature for 15 minutes to allow reaction to take place. Absorbances were measured at a wavelength of 517 nm against methanol as the blank using the UV spectrophotometer. The test was conducted in triplicates and the percentage radical scavenging activity was calculated using the formula as follows:

(1) $$\begin{array}{c} \%RSA=\frac{Abs.\;control-Abs.test}{Abs.control}\times 100\text{,} \end{array}$$

% means the percentage, RSA means the radical scavenging activity, and abs. is the absorbance.

The antioxidant potency was expressed as a value of concentration of *Hypericum revolutum* subsp. *keniense* leaf essential oil that was responsible for 50% inhibition of DPPH radicals (IC_50_). IC_50_ values for the oil and the standard were calculated from the relationship curve of RSA versus concentrations of the respective sample and standard curves. The antioxidant activity levels were classified based on the IC_50_ values, very strong (IC_50_ of less than 50 *μ*g/ml), strong (IC_50_ of between 50 and 100 *μ*g/ml), medium (IC_50_ of between 100 and 150 *μ*g/ml), weak (IC_50_ of between 150 and 200 *μ*g/ml), and very weak (IC_50_ of more than 200 *μ*g/ml) [29].

#### 2.6.2. Ferric Reducing Antioxidant Power (FRAP) Assay

The reducing power of *H. revolutum* subsp. *keniense* essential oil moisture was evaluated by protocol of Moriasi et al. [28] with minor modifications. The essential oil and L-ascorbic acid different concentrations (0.01, 0.1, 1, 10, 100, and 1000 *μ*g/ml) were prepared in methanol. Exactly one-milliliter of the extract or the standard at different concentrations was added into 2.5 ml of 200 mM phosphate buffer (pH 6.6) and 2.5 ml of 30 mM potassium ferricyanide. The resulting mixture was incubated for 20 min at 50°C. Thereafter, 2.5 ml of 600 mM trichloroacetic acid (TCA) was added into the cooled reaction mixture and then centrifuged at 3000 rpm for 15 minutes. A volume of 2.5 ml of the supernatant was mixed with an equal volume of distilled water. Finally, 0.5 ml of 600 mM ferric chloride was added to the mixture. The absorbance of both the standard (ascorbic acid) and the extracts at different concentrations were measured at a wavelength of 700 nm against the blank using a spectrophotometer (Lab tech model double beam UV-Vis). The concentrations of the oil and standard that yielded an absorbance value of 0.5 were determined from the graph of absorbance at 700 nm against extract concentrations and considered as the median effective concentration (EC_50_). Increase in the absorbance indicated high antioxidant activity. In each case, the experiment was performed in triplicate.

#### 2.6.3. Hydrogen Peroxide Radical Scavenging Capacity Assay

The method of Arika et al. [30] with minor modifications was adopted. The *H. revolutum* subsp. *keniense* essential oil and L-ascorbic acid were prepared to yield different concentrations in methanol. Hydrogen peroxide solution (40 mM) was prepared using a phosphate buffer (pH 7.4). One-milliliter of different concentrations of the essential oil/standard (1000–0.01 *μ*g/ml) was added to 0.6 ml of hydrogen peroxide solution and incubated for 10 min at room temperature. The absorbance of the solutions was measured at 230 nm against a blank consisting of phosphate buffer (pH 7.4) only. All the tests were performed in triplicates, and the scavenging activity of hydrogen peroxide was calculated using the formula stipulated by Legas Muhammed et al. [31].

(2) $$\begin{array}{c} \%\;hydr\;ogen\;peroxide\;scavenging\;activity=\left(\frac{A0-A1}{A0}\right)\times 100\text{,} \end{array}$$

where *A*0 is the absorbance of the control reaction and *A*1 is the absorbance in the presence of the samples or standards.

### 2.7. Chemical Analysis of *H. revolutum* subsp. *keniense* Essential Oil Mixtures

#### 2.7.1. Gas Chromatography-Mass Spectrometry (GC-MS) Analysis

Chemical composition of the essential oil isolated from *H. revolutum* subsp. *keniense* leaves and flowers were analyzed by gas chromatography-mass spectrometry SHIMADZU 2010 plus (GC-MS). The GC-MS analyses were performed in three independent repetitions. A 7890A gas chromatography interfaced with mass spectrometer triple quad system fitted with 7693 auto-sampler, Agilent Technologies, and installed with Mass Hunter Workstation Software Version B.05.00 build 5.0.519.0, service pack I © Agilent technologies 2011, for qualitative analysis. A capillary column DB-5, 30 m × 0.25 mm ID, and column film thickness of 0.25 *μ*m composed of 100% dimethylpolysiloxane was used. Helium of 99.9999% purity was used as a carrier gas at a flow rate of 1 ml/min. A sample of essential oil was introduced to the injector at a volume of 1 *μ*l and the split ratio of 10 : 1. The inlet temperature was maintained at 250°C. The oven temperature was programmed at 110°C for 4 min and then increased to 240°C and finally to 280°C at a rate of 20°C for 5 minutes. The total run time for the extract was 40 minutes. The MS transfer line was maintained at a temperature of 250°C, and the source temperature was maintained at 280°C. The mass fragments were analyzed using electron impact ionization at 70 eV and a scan interval of 0.5 seconds, and the fragments were read from 45 to 450 Da, and the obtained data were evaluated using the total ion count (TIC).

#### 2.7.2. Identification of Compounds

The compounds in *H. revolutum* subsp. *keniense* essential oils were identified from chromatograms based on their elution times. The chromatograms of the eluted compounds were deconvoluted and their mass spectra matched with those of the NIST 11 mass spectral database. The NIST 11 database is a fully evaluated collection of electron ionization (EI) mass spectra for various compounds, and it contains MS/MS spectra and GC-data of over 243, 893 spectra of 212, 961 unique compounds with identifications, nearly all with chemical structures. Quantifiable data of *H. revolutum* subsp. *keniense* essential oils contents were computed from the electronic integration of the total ion chromatogram (TIC) peak areas and recorded as area percentage of total which was then considered a percentage composition of the oil.

#### 2.7.3. FTIR Spectroscopy Fingerprinting of *H. revolutum* subsp. *keniense* Essential Oils

Fourier transform infrared spectroscopy analysis of the essential oils was carried out by adopting the method used by Wangia et al. [32] *H. revolutum* subsp. *keniense* essential oils from the leaves and flowers were mixed with spectroscopic grade potassium bromide (KBr) in the ration of 1 : 10 by a mortar and pestle. The resultant fine powder mixture was compressed using a hand press (5 × 10^6^ Pa) in an evacuated die to produce a clear transparent disc of 13 mm diameter and 1 mm thick. The disc was placed in a sample handler and was scanned twenty times at the frequency regions of between 4000 and 450 cm^−1^ and the spectra produced were recorded.

#### 2.7.4. Evaluation of Total Phenolic Content (TPC)

The total phenolic content of *Hypericum revolutum* subsp. *keniense* leaf essential oil extract was determined based on Folin–Ciocalteu reagent assay, as described by Saeed et al. [33] with slight modifications. Different concentrations range from 150 to 4.6875 *μ*g/ml of gallic acid (0.3 ml) were prepared, and they were mixed with 1.5 ml of Folin–Ciocalteu's phenol reagent (10%) by swirling using test tubes. *Hypericum revolutum* subsp. *keniense* leaf essential oil extracts (0.3 ml) were put in a separated test tube and mixed with 1.5 ml Folin–Ciocalteu's phenol reagent (10%) by swirling using test tubes too. Thereafter, 1.5 ml of 7.5% Na_2_CO_3_ solution were made in deionized water and were mixed with 0.3 ml of different concentrations of gallic acid and *Hypericum revolutum* subsp. *keniense* leaf essential oil extracts. The mixtures were incubated for 2 minutes in the dark at room temperature, after which the absorbance was read at 760 nm. The TPC was determined from the extrapolation of gallic acid standard calibration curve. The estimation of the phenolic compounds was carried out in triplicate. The TPC was expressed as milligrams of gallic acid equivalents (GAEs) per g of the sample in dry weight. Results were calculated and expressed as a microgram of gallic acid equivalents (GAEs) per gram extract using the formula described by Siddiqui et al. [34].

(3) $$\begin{array}{c} C=\frac{c\;x\;v\;}{m}\text{,} \end{array}$$

where *C* in mgGAE per gram dry weight of the sample, *c* is the concentration of gallic acid (*μ*g/ml) extrapolated from the curve equation, *v* is the volume in which the sample was diluted (ml), and *m* is the mass of the extract weighed in grams.

#### 2.7.5. Evaluation of the Total Flavonoid Content (TFC)

Total flavonoid content (TFC) of *Hypericum revolutum* subsp. *keniense* leaf essential oil extract was determined by aluminium nitrate colorimetric method described by Cosmulescu et al. [35] with some modifications. In brief, 0.125 ml of 1% *Hypericum revolutum* subsp. *keniense* leaf essential oil extract in methanol and different concentrations of catechin (0.625 to 20 *μ*g/ml) were prepared using test tubes in triplicates. Five percent of 0.075 ml sodium nitrate (NaNO_3_) was added to the test tubes containing the test sample and the different concentrations of the standard (catechin). Thereafter, the mixture was incubated for six minutes at room temperature. Thereafter, the mixtures in the test tubes were incubated for six minutes at room temperature, then followed with the addition of0.15 ml of 10% aluminium nitrate (AlNO3) to each preparation. 0.75 ml of 4 % sodium hydroxide was added to the mixtures, and finally, the volume of each mixture in the test tube was made up to 2.5 ml using distilled water. The absorbance of the reaction mixtures was measured at 510 nm using UV spectrophotometer. The blank was setup by following the same procedure; however, the plant extract was replaced with an equal volume of methanol. All the determinations were carried out in triplicate. The total flavonoid content was expressed as a milligram of catechin equivalents (CEs) per gram dry weight of the extract, respectively, using the formula described by Siddiqui et al. [34].

(4) $$\begin{array}{c} C=\frac{c\;x\;v\;}{m}\text{,} \end{array}$$

where *C* in mgCE per gram dry weight of sample, *c* is the concentration of cathecin in *μ*g/ml that was extrapolated from the curve equation, *v* is the volume in which the sample was diluted (ml), and *m* is the mass of the extract weighed in grams.

### 2.8. Statistical Analyses

Antimicrobial and antioxidant results were presented as the mean ± standard error of the mean. The one-way ANOVA with Turkey's *posthoc* test was performed for the comparison between the means. The statistical analyses were performed using Minitab statistical software version 20 (Minitab, LLC., USA). The data were considered significant when the *P* value was <0.05.

### 2.9. Ethical Considerations

The permission to carry out the study and ethical clearance was granted by the office of the Registrar Research and Extension, Kisii University, on a letter with Ref: KSU/R&E/03/5/529. The methods for the current study were reviewed by Mount Kenya University Scientific and Ethics Review Committee, and a certificate number 875 was granted for research in a letter with Ref: MKU/ERC/1802. The National Commission for Science, Technology and Innovation (NACOSTI) license number NACOSTI/P/21/10472 was also granted for the current research in a letter with reference number 294203.

## 3. Results

### 3.1. Extraction Yield

The dried leaves and flowers had a percentage essential oil yield of 7.5 ± 07% and 3.8 ± 03%, respectively. The essential oil mixture obtained from the leaves was yellow in colour with sweet floral aroma while the one from the flower was brown with sweet fruity aroma.

### 3.2. Antibacterial Activity of *H. revolutum* subsp. *keniense* Essential Oils

In this study, the flower and leaf derived essential oils of *H. revolutum* subsp. *keniense* produced significantly larger growth inhibition zones against the tested bacteria at 100% concentration than at lower concentrations (*P* < 0.05; Table 1). There were no significant statistical differences in growth inhibition of the tested bacteria observed between plates incubated with 12.5% v/v and 25% v/v and 25% v/v and 50% v/v of the flower and leaf oils of *H. revolutum* subsp. *keniense* (*P* > 0.05; Table 1). Notably, the inhibition zones produced by the leaf and flower essential oils against methicillin-resistant *S. aureus* and *H. influenzae* and the flower oil against *A. baumanii* and *S. aureus* were not significantly different from those produced by the standard antibiotic (ciprofloxacin 0.05% w/v) (*P* > 0.05; Table 1).

Besides, the inhibition zones produced by the two types of essential oils at each concentration against all the test bacteria, except methicillin-resistant *S. aureus,* were not significantly different (*P* > 0.05; Table 1). On the methicillin-resistant *S. aureus*, the leaf derived essential oil of *H. revolutum* subsp. *keniense* showed a statistically significant larger inhibition zone than the flower derived essential oil (*P* < 0.05; Table 1); however, the inhibition zones produced by the two oils at all the other concentrations are not significantly (*P* > 0.05) different as shown in Table 1.

A summary of the lowest concentrations of the *Hypericum* leaf and flower essential oils which prevented visible growth (MIC) of the tested bacteria is tabulated (Table 2). The lowest MIC values that were revealed in this study were 15.625 (*μ*l/ml); this was demonstrated by the essential oils from the leaves of *H. revolutum* subsp. *keniense* against methicillin-resistant *Staphylococcus aureus*, *Salmonella enteritidis,* and *Escherichia coli*. Also, the lowest concentration of the oils that was bactericidal (MBC) was 15.625 (*μ*l/ml) for methicillin-resistant *Staphylococcus aureus.* The MBC: MIC ratio was either one (1) or two (2) throughout the experiment.

### 3.3. Antioxidant Activity of *H. revolutum* subsp. *keniense* Leaf Essential Oil Mixture

Three *in vitro* antioxidant activity assay methods were used to demonstrate the ability of the *Hypericum* essential oil from *H. revolutum* subsp. *keniense* to scavenge for free radicals. The methods were based on spectrophotometry, namely DPPH, fE (III) complex, and H2O2 assays.. The percentage radical scavenging activity of the essential oil obtained from *H. revolutum* subsp. *keniense* in the current study revealed IC_50_ values of the oil as more than 1000 µg/ml for DPPH assay, 0.31 *μ*g/ml for FRAP assay, and 12.33 *μ*g/ml in the case of hydrogen peroxide assay.

### 3.4. Antioxidant Activity by DPPH Assay

The three methods of assay had variant IC_50_ values, and this is consistent with literature that explains that different methods utilize different principles [36]. On the basis of the description performed in 2020 by Minsas et al. [29], the DPPH assay revealed very weak antioxidant activity (IC_50_ > 200 *μ*g/ml) when compared to that of L-ascorbic acid which was very string (IC_50_ < 50 *μ*g/ml). The very high IC_50_ values in the DPPH method of assay are ascribed to the inadequacy of the method to the lipophobic systems and given that the essential oil in this case provides for the nonpolar system [37, 38].

#### 3.4.1. Ferric-Reducing Antioxidant Power of the Leaf Essential Oil of *H. revolutum*

The FRAP method a nonfree radical involvement method focusses on the monitoring of the process of reducing of ferric iron (Fe^3+^) to ferrous iron (Fe^2+^). The concentration of the *Hypericum* essential oil under this study was 0–1000 *μ*g/ml, and the calculated ferric reducing antioxidant power that converted half of the Fe^3+^–Fe^2+^ known as IC_50_ was 0.31 *μ*g/ml. As indicated in the results in Table 2, the absorbances of the reaction mixtures containing the leaf oil of *H. revolutum* subsp. *keniense* at concentrations of 0.32 and 1.6 as well as 8.0 and 40.0 (*μ*g/ml) were not significantly different (*P* > 0.05); however, these absorbances were significantly lower than those recorded at concentrations between 200.0 and 1000.0 *μ*g/ml (*P* < 0.05). Furthermore, the standard (L-ascorbic acid) showed a significant concentration-dependent increase in the measured absorbances (*P* < 0.05; Table 3). Notably, the differences between the absorbances of the *H. revolutum* subsp. *keniense* leaf oil and L-ascorbic acid recorded at concentrations of 0.32, 200, and 1000 *μ*g/ml were insignificant (*P* > 0.05); however, at other concentrations, the absorbances recorded by L-ascorbic acid were significantly higher than those recorded by the studied oil (*P* < 0.05; Table 3).

#### 3.4.2. Hydrogen Peroxide Scavenging Activity

The percentage hydrogen peroxide scavenging activity of the *H. revolutum* subsp. *keniense* leaf oil increased significantly (*P* < 0.05) in a concentration-dependent manner, as shown in Table 4. However, the percentage of hydrogen peroxide scavenging activity of L-ascorbic acid recorded between concentrations of 40.0 and 200.0 *μ*g/ml, 1.6 and 8.0 *μ*g/ml, and 0.32 and 1.6 *μ*g/ml, respectively, were not significantly different (*P* > 0.05) whereas that recorded at a concentration of 1000.0 was significantly higher than that recorded at all the other concentrations (*P* < 0.05; Table 4). Notably, the leaf oil of *H. revolutum* subsp. *keniense* had a significantly higher percentage of hydrogen peroxide scavenging activity than L-ascorbic acid at all concentrations (*P* < 0.05; Table 4). The ability of *H. revolutum* subsp. *keniense* leaf essential oil to scavenge for hydrogen peroxide (H_2_O_2_) was studied. The IC_50_ value was calculated to be 12.33 *μ*g/ml, which indicated that such concentration was required to quench half of the available H_2_O_2_ molecules, therefore offering protection from the tissue damage [32, 39].

### 3.5. Chemical Composition of *H. revolutum* subsp. *keniense* Essential Oils

#### 3.5.1. Chemical Composition of Essential Oils Obtained from *H. revolutum* subsp. *keniense* Leaves (HRLEO) and Flowers (HRFEO)

The results obtained from the current study showed that *H. revolutum* subsp. *keniense* essential oils contained complex mixtures of more than 100 compounds (Table 5). The major components of the essential oils that were revealed in the GC-MS spectra of the essential oil mixture that was isolated from the leaves were caryophyllene (22.1%), *α*-farnesene (9.2%), 1-octyl trifluoroacetate (7.9), 2-ethyl-2-methyl-oxirane (6.3%), (Z, E)-3, 7, 11-trimethyl-1, 3, 6, 10-dodecatetraene (4.5%), *α*-Copaene (4.3%), 3- (2-bromoethyl) dihydro-2 (3H)-furanone (3.1%), *α*-Acetobutyrolactone (2.8%), trans-1, 2-bis-(1-methylethenyl), cyclobutene (2.3%), cis-calamenene (2.2%), *α*-Gurjunene (1.8%), caryophyllene oxide (1.8%), and cadina-1 (6), 4-diene (1.5%). Different percentages of essential oil compositions were observed in the essential oil mixture that was derived from flowers and the main chemical components were 7- (1-methylethylidene), bicyclo (4.1.0), heptane (15%), (E)- 2, 7-dimethyl-3-Octen-5-yne (11%), 2, 3, 4-trimethylhexane (10.5%), 2-ethyl-2-methyl-oxirane (8.8%), caryophyllene (8.5%), 1-octyl trifluoroacetate (7.9%), 4, 4-dimethyloxazolidine (6.6%), D-camphene (5.3%), *α*- copaene (3.2%), 2-cyclopropylpentane (1.6%), *β*-Ocimene (1.5%), and undecane (1.5%).

#### 3.5.2. Fourier Transform Infrared Spectra of *H. revolutum* subsp. *keniense* Essential Oils

**Figure 1:**
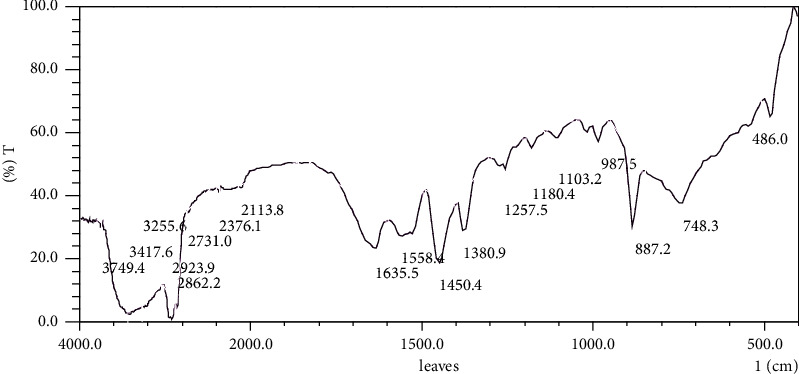
FTIR spectrum of *H. revolutum* subsp. *keniense* leaves essential oil mixture.

**Figure 2:**
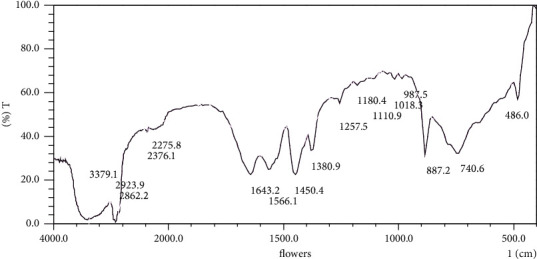
FTIR spectrum of *H. revolutum* subsp. *keniense* flowers essential oil mixture.

The Fourier transform infrared spectra that were obtained from the current study revealed eighteen and seventeen peak values that were interpreted to represent different functional groups. FTIR spectral analysis was used to identify the functional groups of the secondary metabolites that are associated with medicinal values. The region above 1200 cm^−1^ revealed eleven and nine spectral peaks due to the vibration of individual bonds or functional groups of the compounds that were in the essential oils that were obtained from *Hypericum revolutum* subsp. *keniense* leaves and flowers, respectively (Table 6 and Figures 1 and 2). On the other hand, six spectral peaks were observed in the region below 1200 cm^−1^, which is known as the “fingerprint region” that indicates bands due to the vibrations of the complete bioactive molecule. The fingerprint region was notable with many infrared bands. The bands represent different vibrations, including those of O-H, –CH (CH_2_), C=O, C=C-C, C-H, and C-N single bond stretches.

#### 3.5.3. Phenolic Contents of *H. revolutum* subsp. *keniense* Leaf Essential Oil Extract

**Figure 3:**
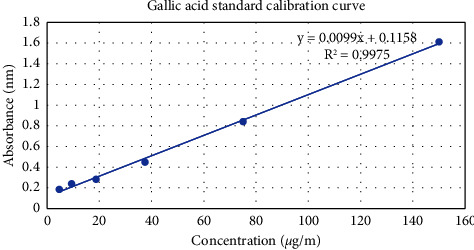
The standard curve of gallic acid.

The phenolic content of the *Hypericum* oil isolated from the leaves of *H. revolutum* subsp. *keniense* was measured using the Folin–Ciocalteu reagent. The results were derived from a calibration curve (*Y* = 0.009*x* + 0.1158, *R*^2^ = 0.9975) of gallic acid (0–150 *μ*g/ml) and expressed in gallic acid equivalents (GAE) per gram dry extract weight (Figure 3). The total phenolic content in the essential oils derived from the leaves was calculated and found to be 130.4 6 ± 10.5 mg GAE/g dry weight.

#### 3.5.4. Flavonoid Content of *H. revolutum* subsp. *keniense* Leaf Essential Oil Extract

**Figure 4:**
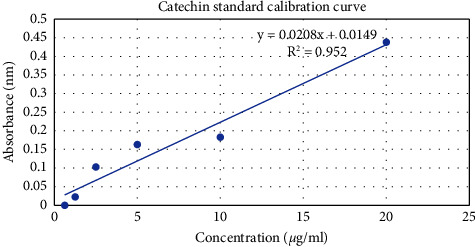
The standard curve of catechin.

The quantitative determination of flavonoids in the essential oils derived from the leaves of total leaves of *H. revolutum* subsp. *keniense* was carried out by aluminium nitrate colorimetric method. The results were derived from the calibration curve (*Y* = 0.0208*x* + 0.0149, *R*^2^ = 0.952) of catechin (0.625–20 *μ*g/ml) (Figure 4) and expressed as mg catechin equivalent (CE) per gram of essential oils dry weight. The calculations revealed that the total flavonoid content essential oil from the dried leaves of *H. revolutum* subsp. *keniense* was 0.911 ± 0.04 mg CE/g dry weight.

## 4. Discussion

The current study provides a report on the antibacterial, antioxidant, and chemical composition of *H. revolutum* subsp. *keniense* essential oil mixture obtained from leaves and flowers for the first time. However, the essential oils from other *Hypericum* species have been reported to have activity against a range of bacteria [40, 41]. The antibacterial activity of essential oils extracts from *H. revolutum* subsp. *keniense* leaves and flowers was interpreted according to Alves et al. [42] as a very high antibacterial activity at 100% undiluted oil. The essential oils demonstrated broad spectrum antibacterial activity against both gram-positive and gram-negative bacteria. The MIC values of both leaves and flowers essential oils extracts less than 8000 *μ*L/ml and can be interpreted according to Voukeng et al. [43] as significant antibacterial activity. The MBC/MIC ratio was found to less than 4 (<4) for the *H. revolutum* subspecies *keniense* leaf and flowers oils extracts against all the assayed bacteria in this study. Therefore, it was interpreted to mean that both the oils had bactericidal effects [25]. More so, the antioxidant activities of the *Hypericum* oil from *H. keniense* subsp. *keniense* in the current study are consistent with the literature of the essential oils from other *Hypericum* species, more especially the exceptionally and highly studied *Hypericum perforatum* [44, 45].

A considerable number of chemical constituents in the essential oils detected in this study were consistent with the ones found in other *Hypericum* species in literature though it was noted that quantities of the composition were different from those of other *Hypericum* species. Some of the major compounds that were identified in the essential oils derived from *Hypericum* leaves and flowers have been reported previously by Crockett and Del Monte et al. [40, 46], and caryophyllene, caryophyllene oxide, ocimene, pipene, spathulenol, myrcene, amorphene, selinene, and undecane were reported in the current study of the essential oils *H. revolutum* susp. *keniense*. However, germacrene D and limonene which are commonly identified in the essential oils of other *Hypericum* species were not identified. The chemical content and percentage variations in the components of essential oils and the variations that were observed in the *Hypericum* oil in the current study are attributed to environmental factors, plant parts, harvesting time, drying procedures, and storage conditions as described by Bergonzi et al. [47]. The antibacterial and antioxidant activities of some of the major compounds that we found in the leaves and flowers of H. revolutum subsp. *keniense* have been reported. For example, caryophyllene and *α*-pinene are indicated to have antibacterial properties in literature [48–51]. D-camphene is also known for its antioxidant activities [48].

The interpretation of FTIR spectra with sixteen bands provide information of varied classes of chemical components of the essential oils. The main classes being alcohols, phenolics, carbonyl ketones, amides, and aldehydes. The band at 3749.4 cm^−1^ for the leave essential oil, indicated the nonbonded O-H stretch of the hydroxyl group, and the broad band between 3315.5 and 3160.2 cm^−1^ and 3417 and –3255.6 cm^−1^ for the leaf and flower essential oils, respectively, depicts the O-H of the hydroxyl group of alcohols. Both the leaf and flower essential oils FTIR spectra revealed the bands between 2923 cm^−1^ and 2862.2 cm^−1^, which are characteristic of asymmetric and symmetric stretch vibrations of C-H groups. Also, the band at 2731.8 represented the C-H group of aldehydes [52]. C-H group of the essential oils was confirmed by the presence of the spectra band at 1450.4 cm^−1^–987.5 cm^−1^ by Burman et al. [53]. The bands at 1635.5 cm^−1^ and 1643.2 cm^−1^ in the FTIR spectra for the leaf and flower essential oils, respectively. These were the characteristic of the presence of C=C that absorbs between 1680–1620 cm^−1^ [54]. The presence of C=C band on the FTIR spectra was associated to the presence compounds with olefinic unsaturated group. In this case, an example is farnesene [55]. The bands at 1558.4 cm^−1^ and 1566.1 cm^−1^ of the leaf and flower essential oil spectra, respectively, represent the presence of stretch vibrations that were indicative of compounds containing carboxylate or carboxylic acid salt that is proposed to originate from 1-octyl trifluoroacetate [56]. The two peaks FTIR spectrum of the H. revolutum subsp. eniense essential oils that appear at ∼1450.4–∼1380.9 cm^−1^ originate possibly from caryophyllene due to the presence of isopropyl and gem-dimethyl groups which give rise to a split umbrella mode [57, 58].

The plant extracts that contain phenolic and flavonoid molecules are known to have antibacterial and antioxidant properties [59]. The antibacterial potency of the phenolic and flavonoid compounds is attributed to their ability to destroy the membrane of bacteria, inhibit the bacterial virulence factors such as enzymes and toxins, and suppress bacterial biofilm formation [60]. On the other hand, these molecules donate hydrogen to neutralize and deactivate the free radicals and have therefore gained popularity for preventing diseases caused by oxidative stress over the synthetic antioxidants [61, 62]. The available literature indicates that some groups of compounds in the essential oils of plants are classified as terpenes, straight‐chain compounds not containing any side chain, phenylpropanoidsand miscellaneous group having varied structures not included in first three groups (sulfuror nitrogen‐containing compounds). Compounds from these groups have antibacterial and antioxidant potency plant extracts that have phenolic and flavonoid molecules, inhibiting bacterial growth as well as preventing cellular damage to the action of free radicles [33, 59, 63, 64]. The extracts and essential oils from *Hypericum* species have demonstrated compounds with antibacterial and antioxidant properties [41, 44, 45, 65]. However, there is no study that has been reported over the essential oils revived from the leaves or flowers of *H. revolutum* subsp. *keniense.*

## 5. Conclusions and Recommendation

Based on the results from the current study, it was concluded that the essential oils obtained from the leaves and flowers of *Hypericum revolutum* subsp. *keniense* had significant antibacterial activity against methicillin-resistant *Staphylococcus aureus, Staphylococcus aureus*, *Acinetobacter baumannii, Salmonella enteritidis, Salmonella typhimurium*, *Pseudomonas aeruginosa*, *Escherichia coli,* and *Haemophilus influenzae.* The findings for antibacterial activity are consistent with the traditional uses of *H. revolutum* subsp. *kenience* in folklore medicine in the treatment of diarrhoea, burns, and wounds. Based on the FRAP and H_2_O_2_ methods of the antioxidant assay, the essential of the leaves have compounds with antioxidant properties. It was presumed that the antibacterial antioxidant potency was due to the compounds such as caryophyllene, *α*-pinene, and D-camphene. It was recommended that research on antioxidant activities and quantification of phenolics and flavonoids be carried out using the flower derived essential oils. Further efforts are needed to assess the wound healing properties and toxicological profiles of the *H. revolutum* subsp. *keniense* essential oils.

## Figures and Tables

**Table 1:** Antibacterial activity of the flower and leaf oils of *H. revolutum*.

Bacterial strain	EOs extract	Concentration and inhibition zone diameter (mm)				Standard (0.05% w/v)
		12.5% v/v	25% v/v	50% v/v	100% v/v	
MRSA	FL	12.00 ± 0.00^b^_A_	14.00 ± 0.58^b^_A_	14.67 ± 0.67^b^_B_	27.33 ± 0.33^a^_A_	29.70 ± 1.20^a^_A_
	LF	13.00 ± 1.00^c^_A_	15.00 ± 0.58^bc^_A_	17.33 ± 0.67^b^_A_	28.00 ± 0.58^a^_A_	29.70 ± 1.20^a^_A_

*S. aureus* (ATCC 12393)	FL	10.33 ± 0.33^d^_A_	14.00 ± 0.58^c^_A_	15.67 ± 0.33^c^_A_	28.67 ± 0.88^b^_A_	31.61 ± 0.27^a^_A_
	LF	11.67 ± 0.33^c^_A_	15.00 ± 0.58^b^_A_	16.67 ± 0.88^b^_A_	32.00 ± 1.00^a^_A_	31.61 ± 0.27^a^_A_

*A. baumannii* (ATTC 19606)	FL	12.00 ± 0.00^da^_A_	13.33 ± 0.33^cd^_A_	14.67 ± 0.33^c^_A_	31.33 ± 0.88^b^_B_	38.30 ± 0.35^a^_A_
	LF	12.00 ± 0.00^c^_A_	13.33 ± 0.33^bc^_A_	15.33 ± 0.33^b^_A_	36.67 ± 0.88^a^_A_	38.30 ± 0.35^a^_A_

*S. enteritidis* (NCTC12023)	FL	10.67 ± 0.33^d^_A_	11.33 ± 0.33^d^_A_	15.00 ± 0.00^c^_A_	22.67 ± 0.33^b^_A_	30.30 ± 0.69^a^_A_
	LF	11.00 ± 0.58^d^_A_	12.00 ± 0.00^d^_A_	15.33 ± 0.33^c^_A_	25.00 ± 0.58^b^_A_	30.30 ± 0.69^a^_A_

*S. typhimurium* (ATCC 14028)	FL	10.00 ± 0.00^d^_A_	12.00 ± 0.58^cd^_A_	14.00 ± 0.00^c^_A_	22.33 ± 0.67^b^_A_	28.63 ± 0.60^a^_A_
	LF	10.33 ± 0.33^d^_A_	12.33 ± 0.67^cd^_A_	14.00 ± 0.58^c^_A_	23.00 ± 0.58^b^_A_	28.63 ± 0.60^a^_A_

*P. aeruginosa* (ATCC 15442)	FL	11.00 ± 0.58^c^_A_	12.00 ± 0.00^c^_A_	13.68 ± 0.33^c^_A_	20.67 ± 0.33^b^_A_	30.00 ± 1.50^a^_A_
	LF	11.33 ± 0.33^c^_A_	12.33 ± 0.33^c^_A_	14.67 ± 0.33^c^_A_	20.67 ± 0.33^b^_A_	30.00 ± 1.50^a^_A_

*E. coli* (ATCC 25922)	FL	11.00 ± 0.00^d^_A_	11.67 ± 0.33^d^_A_	13.67 ± 0.33^c^_A_	20.67 ± 0.33^b^_A_	39.30 ± 0.35^a^_A_
	LF	11.33 ± 0.33^d^_A_	12.67 ± 0.33^cd^_A_	14.00 ± 0.00^c^_A_	21.67 ± 0.33^b^_A_	39.30 ± 0.35^a^_A_

*H. influenzae* (ATCC 49766)	FL	12.33 ± 0.33^c^_A_	14.33 ± 0.33^c^_A_	19.33 ± 0.67^b^_A_	28.33 ± 0.88^a^_A_	29.00 ± 0.00^a^_A_
	LF	12.67 ± 0.33^c^_A_	14.67 ± 0.33^c^_A_	21.33 ± 0.88^b^_A_	29.33 ± 1.20^a^_A_	29.00 ± 0.00^a^_A_

Values are presented as $\bar{x}\pm SEM$ for three replicates. Means with dissimilar superscript lower-case alphabets within the same row are significantly different (*P* < 0.05; one-way ANOVA with Turkey's *post hoc* test), whereas means with dissimilar subscript uppercase alphabets within the same bacterial strain and concentration are significantly different (*P* < 0.05; independent student's *t*-test statistic). Eos: essential oils; FL: flower oil extract of *H. revolutum*; LF: leaf oil extract of *H. revolutum*; MRSA: methicillin-resistant *S. aureus*: standard ciprofloxacin (0.05% w/v) and ZI for the negative control = 0 mm.

**Table 2:** MIC and MBC of *H. revolutum* subsp. *keniense* leaf and flower essential oils.

Microorganism	Plant part	MIC (*μ*l/ml)	MBC (*μ*l/ml)	MBC : MIC ratio
MRSA	L	15.625	15.625	1
	Fl	250	250	

*Staphylococcus aureus* (ATCC 12393)	L	125	250	2
	Fl	125	250	

*Acinetobacter baumannii* (ATTC 19606)	L	62.5	125	2
	Fl	62.5	125	

*Salmonella enteritidis* (NCTC12023)	L	15.625	31.25	2
	Fl	31.25	62.5	

*Salmonella typhimurium* (ATCC 14028)	L	62.5	125	2
	Fl	125	250	

*Pseudomonas aeruginosa* (ATCC 15442)	L	15.625	31.25	2
	Fl	62.5	62.5	1

*Escherichia coli* (ATCC 25922)	L	15.625	31.25	2
	Fl	125	250	

*Haemophilus influenzae* (ATCC 49766)	L	125	125	1
	Fl	125	125	

Fl, flower; L, leaf; MBC, minimum bactericidal concentration; MIC, minimum inhibitory concentration; MBC : MIC, minimum bactericidal concentration : minimum inhibitory concentration ratio; MRSA, methicillin-resistant *Staphylococcus aureus*; *μ*l/ml; microliter per milliliter.

**Table 3:** Ferric-reducing antioxidant power of the *H. revolutum* leaf essential oils.

Concentration (*μ*g/ml)	Absorbance (700 nm)	
	HRLEOs	L-ASA
0.32	0.5247 ± 0.0035^c^_A_	0.5942 ± 0.0176^e^_A_
1.60	0.6065 ± 0.0077^c^_B_	0.7008 ± 0.0141^d^_A_
8.00	0.6225 ± 0.0059^c^_B_	0.8509 ± 0.0060^c^_A_
40.00	0.6684 ± 0.0188^c^_B_	0.8747 ± 0.0068^bc^_A_
200.00	0.9622 ± 0.0406^b^_A_	0.9189 ± 0.0090^ab^_A_
1000.00	1.1569 ± 0.0687^a^_A_	0.9635 ± 0.0162^a^_A_

Values are presented as $\bar{x}\pm SEM$ for three replicates. Means with different superscript lower-case alphabets within the same column are significantly different (*P* < 0.05; one-way ANOVA with Turkey's *posthoc* test), whereas means with different subscript uppercase alphabets within the same row are significantly different (*P* < 0.05; independent student's *t*-test statistic). HRLEOs: *H. revolutum* leaf essential oils; L-ASA: L-ascorbic acid.

**Table 4:** Hydrogen peroxide scavenging activity of the *H. revolutum* leaf oil.

Concentration (*μ*g/ml)	HRLEOs	L-ASA
0.32	20.74 ± 0.86^e^_A_	10.18 ± 0.36^d^_B_
1.60	38.17 ± 0.56^d^_A_	12.93 ± 0.26^cd^_B_
8.00	43.77 ± 0.77^d^_A_	14.97 ± 0.57^c^_B_
40.00	61.54 ± 0.79^c^_A_	21.26 ± 1.30^b^_B_
200.00	72.09 ± 3.56^b^_A_	22.59 ± 0.47^b^_B_
1000.00	85.42 ± 1.52^a^_A_	75.24 ± 1.08^a^_B_

Values are presented as $\bar{x}\pm SEM$ for three replicates. Means with different superscript lower-case alphabets within the same column are significantly different (*P* < 0.05; one-way ANOVA with Turkey's *posthoc* test), whereas means with different subscript uppercase alphabets within the same row are significantly different (*P* < 0.05; independent student's *t*-test statistic). HRLEOs: *H. revolutum* leaf essential oils; L-ASA: L-ascorbic acid.

**Table 5:** Chemical composition of essential oils obtained from *H. revolutum* subsp. *keniens*e leaves (HRLEO) and flowers (HRFEO).

No.	RT (min)	Compound	% content of total	
			HRLEO	HRFEO
1	1.7	2-methyl-butane	0.1	0.1
2	1.71	N-Dimethylaminomethyl-tert.-butyl-isopropylphosphine	0.2	0
3	1.72	N, N-dimethyl-3-buten-1-amine	0.3	0
4	1.9	4, 4-dimethyloxazolidine	0	6.6
5	2.0	3-(2-bromoethyl) dihydro-2 (3H)-furanone	3.1	0
6	2.02	*α*-acetobutyrolactone	2.8	0
7	2.1	2-ethyl-2-methyl-oxirane	6.3	8.8
8	2.3	1-octyl trifluoroacetate	7	7.9
9	2.5	2-chlorohexane	1.3	0
10	2.5	2-cyclopropylpentane	0	1.6
11	5.4	5-methyl-2-(1-methylethyl)-1-hexanol	0	0.1
12	6.3	Nonane	0.9	0
13	6.4	2, 3, 4-trimethylhexane	0	10.5
14	7.3	*α*.–pinene	2.3	0
15	7.4	(E)- 2, 7-dimethyl-3-octen-5-yne	0	11
16	7.8	L-camphene	0	0.4
17	7.9	2, 4-thujadiene	0	0.1
18	8.6	*β*-pinene	0.2	0
19	8.7	D-camphene	0	5.3
20	8.8	*β*-myrcene	0.1	0.6
21	9.0	Decane	0	0.1
22	9.4	Thujene	0	0.1
23	9.7	*α*-terpinene	0	0.3
24	10.2	trans-1, 2-bis- (1-methylethenyl) cyclobutane	2.3	0
25	10.3	7- (1-methylethylidene) bicyclo (4.1.0) heptane	0	15
26	10.6	*β*-ocimene	0.1	1.5
27	11.0	*γ*-terpinene	0.1	0.4
28	11.9	3-methyl-6-(1-methylethylidene)-cyclohexene	0.1	0.4
29	12.1	Undecane	0.1	1.5
30	12.2	3-nonanol	0	0.1
31	13.1	exo-fenchol	0	0.1
32	14.5	2-pentadecyn-1-ol	0	0.1
33	15	Pinocamphone	0	0.1
34	15.5	L-*α*-terpineol	0	0.1
35	15.6	Myrtenal	0	0.1
36	19.7	*α*-cubebene	0.2	0
37	20.2	*α*-bulnesene	0.1	0.1
38	20.4	1, 2, 3, 4, 4a, 7-hexahydro-1, 6-dimethyl-4- (1-methylethyl)-naphthalene	0	0.2
39	20.41	Cyclosativene	0	0.2
40	20.43	*α*-ylangene	0.3	0.4
41	20.6	*α*-copaene	4.3	3.2
42	21.5	*α*-gurjunene	0.8	0.5
43	22.1	Caryophyllene	22.1	8.5
44	22.2	*β*-copaene	0.9	0.1
45	22.4	epi-*β*-caryophyllene	1.4	1
46	22.5	Selina-5, 11-diene	0	0.1
47	22.6	(1R, 4aS, 8aR)-1, 4a-dimethyl-7-(prop-1-en-2-yl)-1, 2, 3, 4, 4a, 5, 6, 8a-octahydronaphthalene	0.1	0
48	23.0	(1R, 9R, E)-4, 11, 11-trimethyl-8-methylenebicyclo [7.2.0] undec-4-ene	0.6	0
49	23.4	(1S, 4aR, 8aS)-1-isopropyl-7-methyl-4-methylene-1, 2, 3, 4, 4a, 5, 6, 8a-octahydronaphthalene	1.4	0
50	22.9	(1Z, 4Z, 7Z)-1, 5, 9, 9-tetramethylcycloundeca-1, 4, 7-triene	0.8	0.3
51	23.2	Cadina-1 (6), 4-diene	1.5	0
52	23.3	*γ*-amorphene	0	1.2
53	23.4	(1S, 4aR, 8aS)-1-isopropyl-7-methyl-4-methylene-1, 2, 3, 4, 4a, 5, 6, 8a-octahydronaphthalene	1.2	0.7
54	23.6	(Z, E)-3, 7, 11-trimethyl-1, 3, 6, 10-dodecatetraene	4.5	0.1
55	23.7	*δ*-selinene	0	0.1
56	23.8	[1aR- (1a*α*, 7*α*, 7a*β*, 7b*α*)]-1a, 2, 3, 5, 6, 7, 7a, 7b-octahydro-1, 1, 4, 7-tetramethyl-1H-cycloprop[e]azulene	0.9	0
57	23.8	guaia-1 (10) 11-diene	0	0.7
58	24.0	*α*-farnesene	9.2	0.7
59	24.7	cis-calamenene	2.2	0
60	24.8	11-hydroxy-11-methyl-tricyclo [4.3.1.1(2, 5)] undecan-10-one	0.1	0
61	24.9	Cubenene	1	0
62	24.92	*α*-amorphene	0	0.2
63	25.0	(4aR, 8aS)-4a-methyl-1-methylene-7- (propan-2-ylidene) decahydronaphthalene	0	0.2
64	25.04	(1S, 4aR, 8aS)-1-isopropyl-4, 7-dimethyl-1, 2, 4a, 5, 8, 8a-hexahydronaphthalene	0.2	0.2
65	25.1	9-methoxycalamenene	0	0.3
66	25.13	*α*-maaliene	0	0.3
67	25.2	*α*-calacorene	0.3	0
68	25.5	(E)-3, 7, 11-trimethyl-1, 6, 10-dodecatrien-3-ol	1.4	0.2
69	25.6	Ledene oxide-(II)	0	0.1
70	25.62	cis-(-)-2, 4a, 5, 6, 9a-hexahydro 3, 5, 5, 9 tetramethyl (1H) benzocycloheptene	0	0.1
71	25.64	Isoaromadendrene epoxide	0.1	0
72	25.7	(1aR, 4S, 4aR, 7R, 7aS, 7bS)-1, 1, 4, 7-tetramethyldecahydro-1H-cyclopropa[e]azulen-4-ol	0.1	0.1
73	26.0	1, 1, 4, 7-tetramethyldecahydro-4aH-cyclopropa[e]azulen-4a-ol	0.2	0.2
74	26.3	(5S, 6R, 7S, 10R)-7-isopropyl-2, 10-dimethylspiro [4.5] dec-1-en-6-ol	0.9	0
75	26.4	Caryophyllene oxide	1.8	0.6
76	26.5	5-azulenemethanol	0.3	0
77	26.6	[1aR-(1aalpha, 4beta, 4abeta, 7alpha, 7abeta, 7balpha)]-decahydro-1, 1, 4, 7-tetramethyl-1H-cycloprop[e]azulen-4-ol	0.2	0.2
78	26.7	Bicyclosesquiphellandrene	0.1	0
79	26.8	Cycloheptane, 4-methylene-1-methyl-2-(2-methyl-1-propen-1-yl)-1-vinyl-	0.1	0
80	26.83	Ledol	0	0.1
81	26.9	Globulol	0.6	0.2
82	2.70	(1R, 3E, 7E, 11R)-1, 5, 5, 8-tetramethyl-12-oxabicyclo [9.1.0] dodeca-3, 7-diene	0.2	0
83	27.1	2-(bromomethyl)-2-adamantanol	0.1	0
84	27.4	Di-epi-1, 10-cubenol	1.8	0
85	27.5	*β*-guaiene	0.3	0
86	27.6	10-epi-*γ*-eudesmol	0.5	0
87	27.62	Cryptomeridiol	0.1	0
88	27.7	Epicubenol	0.9	0.4
89	27.73	*τ*-cadinol	0.8	0.2
90	27.8	*α*-muurolol	0.3	0.2
91	27.9	Cyclocopacamphan-12-yl methyl ether A	0.1	0
92	28.0	Isoaromadendrene epoxide	0	0.1
93	28.1	*τ*-muurolol	0.4	0
94	28.13	2-(4a, 8-dimethyl-2, 3, 4, 5, 6, 8a-hexahydro-1H-naphthalen-2-yl) propan-2-ol	0.3	0
95	28.2	Bulnesol	0.2	0
96	28.4	Trans-Z-*α*-bisabolene epoxide	0.3	0
97	28.42	Heptadecane	0	0.1
98	28.6	8-isopropyl-1, 5-dimethyltricyclo [4.4.0.02.7] dec-4-en-3-one	0.2	0
99	28.9	(1R, 7S, E)-7-isopropyl-4, 10-dimethylenecyclodec-5-enol	0.2	0
100	29.1	(E, E, E)-3, 7, 11, 15-tetramethylhexadeca-1, 3, 6, 10, 14-pentaene	0.1	0
101	29.3	Trans-valerenyl acetate	0.1	0
102	29.5	3-(1-methoxy-2-methyl-2-propanyl)-1, 2, 4, 5-tetramethylbenzene	0.1	0
103	29.8	(-)-spathulenol	0.3	0
104	30.1	3, 4, 4a, 5, 6, 7-hexahydro-2H-naphthalen-1-one	0.1	0
105	32.4	Di-2-methylpropyl phthalate	0.1	0
106	32.8	2, 6, 10, 15-tetramethylheptadecane	0	0.3
107	36.8	Heneicosane	0	0.1
108	37.2	Phytol	0.1	0

HRLEO, *H. revolutum* essential oil; HRFEO, *H. revolutum* essential oil; RT, retention time in minutes; % content of total, mean percentages composition of the oils from triplicate experiments; The 0 % value of content of the total in the table represents any percentage content of the total that was less than 0.1%.

**Table 6:** Assignment of FTIR frequencies of *H. revolutum* subsp. *keniense* essential oils.

Peak number	Frequency peak value of *H. revolutum* subsp. *keniense* essential oils		Assignment of the functional group
	HRLEO	HRFEO	
1	3749.4	Not detected	O-H stretch
2	3315.5–3160.2	3417–3255.6	O-H stretch
3	2923	2923	A symmetric stretching of –CH (CH_2_) vibration
4	2862.2	2862.2	Symmetric stretching of –CH (CH_2_) vibration
5	2731.8	Not detected	O-H stretch
6	2376.1–2113.8	2376.1–2275.8	C-H bend
7	1635.5	1643.2	C=C, stretch
8	1558.4	1566.1	Carboxylate
9	1450.4	1450.4	C-H bend
10	1380.9	1380.9	O-H bend, alcoholic group
11	1257.5	1257.5	CN, aromatic primary amine
12	1180.4	1180.4	C-O
13	1103.2	1110.9	CH_3_
14	987.5	987.5	C-H Alkene
15	887.2	887.2	C-H
16	748.3	740.6	-(CH2) n

## Data Availability

The statistical data used to support the findings of this study are included within the article.
